# LC-DEX: Lightweight and Efficient Compressed Authentication Based Elliptic Curve Cryptography in Multi-Hop 6LoWPAN Wireless Sensor Networks in HIP-Based Internet of Things †

**DOI:** 10.3390/s21217348

**Published:** 2021-11-04

**Authors:** Balkis Bettoumi, Ridha Bouallegue

**Affiliations:** 1 Innov’COM Laboratory, Sup’Com, University of Carthage, Technological City of Communications, Route de Raoued Km 3,5, Ariana 2083, Tunisia; ridha.bouallegue@supcom.tn; 2 Doctoral School of Engineering Sciences and Techniques, National Engineering School of Tunis (ENIT), University of Tunis El Manar, BP 37 Le Belvedere, Tunis 1002, Tunisia

**Keywords:** Lightweight Authentication, Wireless Sensor Network, Internet of Things, 6LoWPAN, CoAP, RPL, mesh-under, energy consumption, opportunistic mode

## Abstract

The high level of security requirements and low capabilities of constrained devices that are connected to the Internet of Things (IoT) constitute a new challenge in terms of proposing an authentication solution that deals with the problem of energy constraints. The Host Identity Protocol Diet EXchange (HIP DEX) is primarily designed to be suitable for constrained devices and designed to be resistant to Denial of Service (DoS) and man-in-the-middle (MITM) attacks. In this paper, we propose an efficient saving energy solution to secure end-to-end (E2E) communications based on the compression of the IPv6 over Low Power Wireless Personal Area Networks (6LoWPAN) header for HIP DEX packets. We implement our solution in an IoT based-WSN over Constrained Application Protocol (CoAP) in the application layer and Routing Protocol for Low power and lossy networks (RPL) in the routing layer. We also propose a novel distribution model that minimizes the number of signaling messages. Both proposed compression and distribution models for HIP DEX combined with an original implementation of an opportunistic association establishment of the handshake, constitute an efficient security solution for IoT. We called our solution Lightweight Compressed HIP DEX in the IoT (LC-DEX).

## 1. Introduction

Wireless Sensor Networks based-IoT is becoming a part of our daily life and activities because of the emerging technologies and smart applications that deal with human necessities. In the future Internet, there will exist more connected devices than there are humans on our planet. These IoT devices, the subject of our research, are defined by two main characteristics: (1) Having the possibility of connecting to the Internet; (2) Integrating technologies such as firmware, sensors, actuators and even executing embedded Operating Systems like Linux and Contiki. Applications in the IoT are of great variety, but can be divided into two principal types in relation to the device’s state: mobile device or stationary device. Both types of IoT devices have the same major low energy and computation capabilities. In addition, both can communicate together. The IoT targets at enabling several new technologies, such as intelligent Wireless Sensor Networks (WSNs), smart cities, smart homes, mobile-health (m-health) systems and industrial IoT. Thereby, new types of communications occur [1] such as Man-to-Thing (M2T), Machine to Machine (M2M), Ubiquitous Sensor Networks (USNs), and so on. These technologies have most recently been established as Cyber-Physical Systems (CPSs) [2]. WSNs are considered as the most based-IoT unit [3] that we qualify as the IoT-backbone. For these reasons, research is focused on lightening existing protocols that are compliant with IP-based standards, to be suitable with WSN constraints. Being interested in studying security protocols and techniques in IoT, we can conclude that the most adaptation IP-based protocols includes Datagram Transport Layer Security (DTLS) [4], HIP DEX [5], a minimal Internet Key Exchange (IKEv2) [6] and Extensible Authentication Protocol (EAP) [7]. HIP DEX was standardized to be suitable with low power and resources constrained devices in IEEE 802.15 networks and used as a keying material in the MAC layer. While the existing authentication and key generation protocols are adapted to be suitable for constrained environments, HIP DEX was standardized to deal with the constraints of IoT and is also strong against Denial of Service attacks by its puzzle mechanism. The most difficult challenge is to propose an authentication solution that perform both high level of security in the connection establishment and low-energy consumption by the IoT device. HIP-based solutions constitute a revolution in this field since they provide multihoming and mobility of devices by decoupling the double function of IP addresses as geographical locator and device identifier. Several works focused on proposing IP-based solutions with compression techniques, minimizing exchanged messages to optimize the communication overhead, and on computation acceleration or distribution techniques. So, privacy and individual information related to mobility and health status are more critical when WSNs become opened to the Internet. In our previous works [8], we demonstrated the efficiency of HIP DEX in establishing a M2T secure association, and in [9], we proposed an efficient compression header of HIP DEX protocol over 6LoWPAN in a T2T architecture. Evaluation results in terms of transmission delays during the handshake minimize considerably the communication overhead between the communicating peers (the initiator and the responder). Obtained results, in [9], are exploited in this paper to evaluate the energy consumption during the handshake. This work is an extended version of our recent conference paper [9] with amelioration of the proposed compression technique. We implemented HIP DEX as a Linux daemon that could be executed under any sensor node that supports either Linux and Contiki OS. This implementation was coded with the best support of IPv4, IPv6, and 6LoWPAN protocols and tested on mesh-under network architecture. Obtained results are performed by real experiments in FIT IoT-LAB, an IoT-dedicated platform in France [10]. As demonstrated in Section 6.3, the overall E2E transmission delay performed by LC-DEX is only 0.32 s, comparing with previous Elliptic Curve Cryptography (ECC) solutions. Regarding energy efficiency, LC-DEX costs only 5.55 mJ for the hole handshake process. As a complementary extension work of our conference paper, we contribute by the following in this present work.

Compared with the previous HIP-based solutions, the innovations and contributions of LC-DEX are as follows:

1. An optimization of our previous proposed compression of HIP DEX protocol header [9] over an adaptable 6LoWPAN compression header;
2. A minimization of the optional signaling packets (NOTIFY and CLOSE_ACK) and a proposition of a replacement technique in a novel distribution model;
3. An efficient minimization in the overall E2E transmission delays of the HIP DEX handshake;
4. An important reduction on the computational and communication energy costs of the handshake;
5. Alleviation of HIP peers from the scalar multiplication of the key generation by the integration of a trust third party;
6. Proposition of a new lightweight security association establishment scheme for LC-DEX;
7. Proposition of a HIP DEX opportunistic mode during handshake packets’ exchange with the introduction of a Domain Name System service;
8. Studies of the security threats and requirements of the proposed solution.

This paper presents the following sections: We present the problem statement in Section 2. In Section 3, we present the background of this work regarding the authentication in IoT networks based on HIP solutions and the improved compression model in comparison with the obtained results in the conference paper. Section 4 is dedicated to the proposed minimization in the signaling packets and the novel proposed distribution scheme. The evaluation and experimental results are presented in Section 6. We give an overview of the memory requirements of our implementation in Section 7. The security considerations are explained in Section 8. Section 9 presents the discussion of the results. Finally, we end this work with the conclusion and future work in Section 10.

## 2. Problem Exposure and Motivations

The authentication process is more critical in a constrained network environment than in a traditional network, since there are many types of IoT applications that can damage the life of a human if the authentication phase fails. In this field, we can give an example of a health sensor that is implanted on the heart of a patient and that must send sensitive information to the medical server. We must not forget that IoT also connects the astronomic application to the appropriate working group. Any error in the authentication may cause hard spoilage in monitoring applications and/or satellites. Hence, for an efficient E2E security establishment association on IoT, it is crucial to propose a solution that can secure communication in (M2T) and in Thing-to-Thing (T2T) architectures. HIP DEX is the most promising protocol designed especially for the constrained environment. HIP DEX is characterized by the following encouraging features:

- The separation of the identity and the physical location of the communicating peers is the strength of the HIP-based solution that facilitates end-users mobility and ensures a good level of location privacy. HIP separates the end-point identifier and locator roles of IP addresses. These qualities are highly recommended in many IoT deployments, as in health care and military applications;
- The number of exchanged messages to establish a key agreement mechanism is only four messages, which is not the case for the other security protocols;
- HIP is adopted as a lightweight solution for the WSN [8,9,11,12,13];
- The use of HIP as a Keying material and IPsec for the tunneling for securing the communication between the two parts. This guarantees the security of communication in the network layer (coupling IPsec with HIP) and, as a consequence, securing all types of transport traffic layer (UDP or TCP);
- The protocol is designed to be resistant to Denial of Service (DoS) and man-in-the-middle attacks [5].

The above-cited features are generic for both types of HIP protocol: HIP BEX (HIP Base EXchange) and HIP DEX. HIP BEX has too-heavy cryptographic operations that could not be supported by resource constraint devices such as mobile devices and base stations. To address constrained devices better, the use of ECC has been proposed to be used with HIP [11]. HIP introduces a cryptographic namespace of stable host identities (HIs) between both network and transport layer. Besides enabling multihoming and mobility, the cryptographic HIs ensure a mutual authentication Diffie–Hellman key exchange, in which the generated symmetric-key usually protects an IPSec association. Consequently, ECC is the best computational technique for generating key material with few operations in comparison with asymmetric cryptographic algorithms such as RSA (Rivest–Shamir–Adleman) without losing security level. The relevant differences that exist between HIP BEX and HIP DEX are the total cancellations of the signature scheme in HIP BEX and the combination of the DH key generation with an ECC variant (ECDH). Indeed, the HIP DEX protocol [5] is primarily designed for resolving computation or memory-constrained sensor/actuator devices. The constrained IP-enabled networks, such as 6LoWPAN networks, constitute the backbone of research targeting the Internet of Things. In this context, we are studying, by way of real experiments, the efficiency of HIP DEX in the handshake process in terms of packets’ transmission delays in a 6LoWPAN-WSN IoT.

## 3. Background and Related Works

### 3.1. Authentication Goals in IoT

Authentication in IoT is similar to that in the current Internet but, particularly, it concerns the following:

- Devices with low memory and computational capabilities;
- New applications and services are provided between users and devices in IoT;
- Solid nodes and secure communication for users;
- IoT devices can be integrated into all aspects of existing domains;
- Security of communications between nodes in WSN is critical because it is difficult or even impossible for human intervention after the deployment of sensors;
- Devices are battery-powered in general, and embedded applications must have low computational costs to save energy;
- Real-time applications in IoT require a high level of security with lightness in execution;
- IoT domains become very wide and distance between the sensor node and/or servers must not have a consequence on the communication overhead.

In the following section, we focus on detailing the existing HIP-based solution that is the subject of this paper, then we provide an overview of several works of proposed authentication solutions in IoT.

#### 3.1.1. HIP-Based Solutions

In this section, we detail the principle techniques provided with authentication solution based on HIP protocol. We also provide an overview and a comparison between studied previous HIP-based authentication solutions discussed in this section (see Table 1). HIP [14] protocol is an alternative solution to IKE/IPsec. HIP BEX is based on the cryptographic host identities for mutual authentication, and it establishes dynamic security associations between HIP peers on the Internet. HIP BEX uses the Diffie–Helman key exchange. The handshake is composed of four messages. The public key is serving as a host identifier that each HIP peer should have, and its counterpart private key is known and used only by its legitimate owner. This pair of keys is useful for identification and authentication aims. Once HIP security SA is established, the end-parties can start communicating data, securely, using ESP [15]. HIP BEX handshake involves heavy asymmetric cryptographic operations using certificates and for this reason, it cannot be supported as it is by constrained small devices. In [5], the authors propose to use HIP DEX as an alternative variant of HIP BEX by reducing its computational cost and introducing elliptic curve cryptography in Diffie-Hellman policy ECDH (Elliptic Curve Diffie-Hellman). Only one public key is used to compute the session key and to identify the communicating HIP peer. Some researchers are focused on improving the efficiency of HIP DEX to be compatible with the most constrained sensor nodes. In [16], the authors propose **LHIP (Lightweight HIP)** solution that keeps the same structure of HIP BEX messages and provides a security mechanism based only on hash functions to provide the integrity of the exchanged messages. Security features are maintained and communicated over the exchanged messages but ignored by the pairs. LHIP is weak because it omits primordial security mechanisms as mutual authentication and key exchange. Even if HIP (both HIP BEX and Diet-HIP) is based on a few signaling messages to establish a secure association between two peers on the Internet, these messages are very long and the expensive cost in terms of energy consumption cannot be ignored and also the fragmentation/reassembly rate resulting from the communication of similar messages within a 6LoWPAN network. To resolve this problem, the authors in [17] propose a compressed layer for HIP DEX named **Slimfit**. The compression concerns some fields in the HIP header and signaling parameters for the reduction of the communication cost. The evaluation of the proposed compression demonstrates a lower packet fragmentation rate and less energy consumption. Nevertheless, the solution has compatibility problems with the standard HIP in addition to the heavy computational cost that is relatively important, when it is executed by constrained sensor nodes. Besides, the given compression is not standardized according to the 6LoWPAN compression rules. Relating to compression based solutions, **CD-HIP** [18] combines both Compression (**C-HIP**) and Distribution (**D-HIP** [19]) techniques. This solution shows its performance on the handshake and the delegation of the heavy cryptographic computation to the third party (a collaborator). **Sahraoui and Bilami’s** solution (CD-HIP) shows its performance against the previous works. Nevertheless, their solution is based on HIP BEX that is not designed for constrained devices and even with the use of the third party because it has an energetic cost (communication and computation) that is significantly high. In [20], Ben Said et al. focused their work on a collaborative key establishment solution for IoT devices. Consistently, a constrained sensor node delegates its heavy cryptographic operation to a neighborhood that is less constrained. In the same work, the authors show the possibility of integrating the collaborative proposition with TLS handshake and IKE key generation protocols. Even though establishing a secure End to End connection between two unknown sensors in heterogeneous networks has a great probability of establishing insecure communication. **CHIP** for Collaborative HIP [21] presents a new protocol that is the combination of some features of HIP BEX and HIP DEX. Accordingly to this solution, the authors consider that the responder is the most constrained device than the initiator. That is why they consider delegating the most cryptographic operations of the responder to the proxies but for the initiator only some operations. CHIP includes several proxies on the initiator side and another number of proxies on the responder side. Augmenting the number of third parties increases the security risks and makes it almost impossible to fix faults in the case of the failure of one of the proxies.

#### 3.1.2. Existing M2M-IoT Mutual Authentication Solutions

We detail, in this section, existing IoT solutions that are not based on HIP protocol. The authors of [24] propose a new mutual authentication protocol, based on public-key encryption, for IoT. They demonstrate that the emulated solution is efficient on computational cost and applies to device-to-device communication and device-to-server communication for Smart City applications. The efficiency of the lightweight public-key encryption scheme is studied only for communication cost, while evaluating the computation overhead for resource-constrained IoT devices is a crucial criterion that must be considered for such solutions. Besides, the authors conducted their evaluation with a 112-bits security level when comparing with ECC solutions. Nevertheless, for a high level of security with an ECC authentication solution, the recommended NIST curve is NIST P-256 [25]. The work in [26] presents an Identity-Based Cross-Domain Authentication Scheme for the Internet of Things (IRBA ). IRBA is an identity-based cross-domain authentication scheme for the Internet of Things. Authors replace the traditional certificate of authority with the Blockchain as a decentralized trust anchor and use the identity-based self-authentication algorithm to replace the traditional PKI. Their evaluation is done separately for two scenarios: intra-domain access and inter-domain access. For authenticating the user, authors define an algorithm based on identity and without the need of a trusted third party. IRBA is an IoT authentication solution that is not suitable for communications in an architecture based only on constrained devices since its implementation involves powerful servers (PC with Intel-Core i5 6300 HQ CPU (2.30 GHz), 16 GB). Relating to Industrial IoT (IIoT), the contribution in [27] defines an IoT mutual authentication solution for smart devices in IIoT applications. The smart device must register on the server, as a first step. The second step is mutual authentication between the smart device and the router. During these two steps, five messages must be exchanged between the involved components (the smart device, the authentication server, and the router). This high number of exchanged messages increases the communication overhead. In addition, the smart sensor sends a pre-shared key set to each router on the network, but authors do not define any mechanism to verify the identity of the smart device by the router such as the implementation of Access Control Lists. The same problem is posed when the smart device sends its unique ID to the server. This solution is not resistant against node capture of the router, flooding attack, or data freshness attack. Besides, the authors do not give an overview of the communication and computation costs for different values of the setting parameters (only evaluation for a fixed 128 value for all the parameters) and there is not any comparison with related works. Remaining in IIoT, the proposed authentication protocol Lightweight Authentication and Key Distribution (LAKD) for M2M communication [28] minimizes the number of exchanged messages from five to four, comparing with [29]. LKAD is strong against many network attacks and performs better costs in terms of communication and computation comparing with previous works. IIoT is out of the scope of this paper.

### 3.2. Authentication with HIP DEX Protocol

**Figure 1:**
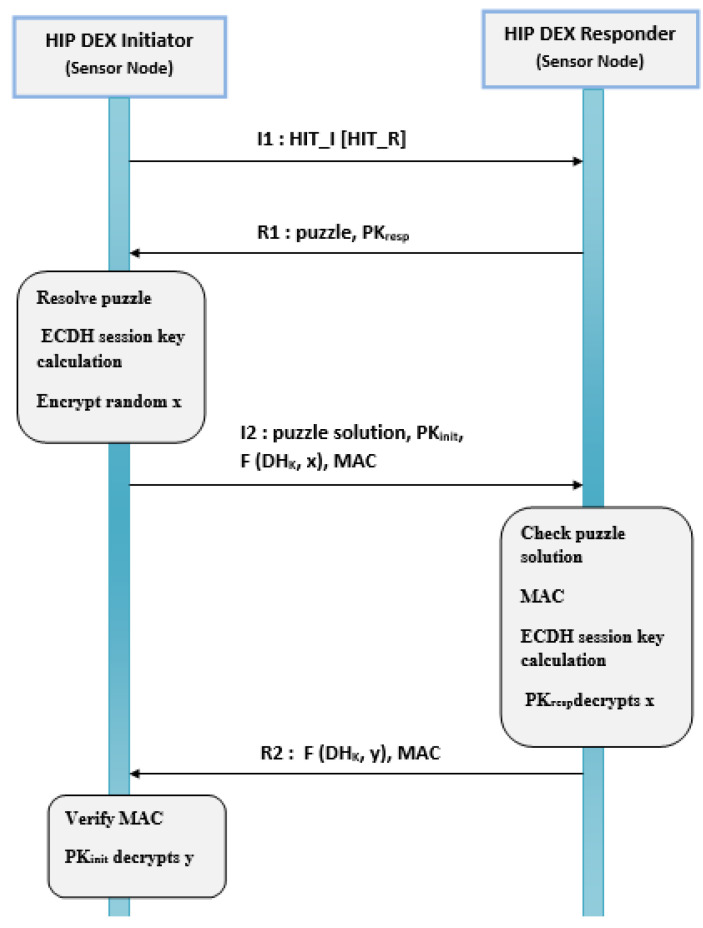
Handshake exchanged messages of standard HIP DEX.

HIP BEX is designed to be used in authentication between a client and a base station and is not suitable for IoT and constrained environments. HIP DEX preserves general HIP architecture and protocol semantics. Precisely, it redefines the cryptographic namespace based on the network device’s public key fixed as a Host Identity (HI). As a consequence, a new layer, placed between the network and the transport layer, is implemented based on the defined namespace. Another fundamental parameter is the Host Identity Tag (HIT) calculated using HI to represent its stable format. It serves as a fixed device identifier at this layer on the network stack. It represents a marked characteristic of the HIP protocol by allowing the mobility of devices without being dependent on IP addresses’ double role of identification and geographical location. HIP DEX protocol aims to permit secure communication of two entities based on cryptographic operations to guarantee a secure communication link between hosts in a constrained environment. The first entity is known as the initiator and the second one the responder. Then, HIP DEX is deprived of the calculation of transitory Diffie–Hellman keys and digital signatures. As a substitute, it defines a light handshake phase based on static DH for mutual key agreement. HIP DEX defines four types of messages (I1, R1, I2, R2). I1 is the first message that contains the sender host identity tag (HIT_I) and a receiver host identity tag (HIT_R) considered as an optional field [5]. The second message, R1, contains a puzzle (a cryptographic challenge) that must be resolved by the Initiator like in HIP BEX, and the public key $PK_{resp}$. The initiator and responder produce DH Key by performing Elliptic Curve Diffie–Hellman (ECDH) for the session key generation using public key (PK) values. The solution to the puzzle must be sent in the third message I2 that contains the $PK_{init}$ and the key parameters F($DH_{k}$, x). The random values x and y are, respectively, the initiator’s and responder’s private keys used to the final session secret key computation. The reinforcement of the security in HIP DEX against the falsification of messages is guaranteed in I2 after the definition of the MAC (Message Authentication Code) value. The fourth message R2 also contains the responder’s key parameters F($DH_{k}$, y). The ECDH generated key is used to encrypt the secrets (x, y), used after that to calculate the session key. Figure 1 presents the standard exchanged messages during the handshake in HIP DEX.

### 3.3. Overview of 6LoWPAN Compression Solutions

Packet compression is a fundamental technique adopted as an adaptation for several protocols in the context of constrained networks. So, minimizing packet length has a direct impact in minimizing the energy consumption of a constrained device in processing and overcoming the big differences in capabilities of IP networks and WSNs integrated into IoT. Especially, when the problem is related to traffic admission ability and the maximum possible authorized size of the protocol data unit in both networks. IPv6 requires the maximum transmission unit (MTU) to be at least 1280 octets. In contrast, IEEE 802.15.4’s standard packet size is 127 octets. A maximum frame overhead of 25 octets spares 102 octets at the media access control layer. An optional but highly recommended security feature at the link-layer poses an additional overhead. For example, 21 octets are consumed for AES-CCM-128 leaving only 81 octets for upper layers. In this context, the 6LoWPAN adaptation layer specifies the rules of the compression and fragmentation of IPv6 datagrams so that they could safely fit in Internet-integrated WSNs. The 6LoWPAN standard is defining header compression and fragmentation of IPv6 datagrams over IPv6-connected WSNs. The compression process consists of IP Header Compression (IPHC) and Next Header Compression (NHC). Thus, all operations of compression/decompression and fragmentation/reassembly of all exchanged IPv6 packets in both directions (incoming/outgoing) are performed by the 6LoWPAN Border Router (6BR). The compression technique has many important advantages and interesting features, among which we cite:

- Minimal communication cost: reduced length of compressed packets has as consequence a lower amount of formation will be communicated;
- Lower energy consumption for the communication phase;
- Minimal packet fragmentation rates: the reduced sizes of the exchanged packets help to reduce the fragmentation frequency;
- Gain in storage space;
- Optimization of the used throughput: the removal of unnecessary bytes of information from a packet’s header, data throughput will be eventually improved because significant bytes in the message will be free to contain the application data.

The IPv6 protocol has a 40 bytes header that is composed of a substantial amount of compressible fields: IPv6 source and receivers addresses, length, and many others [30]. These fields are the only ones that can be compressed or removed. So, IPv6 nodes are hierarchically assigned 128 bit IP addresses, through an arbitrary length network prefix. IEEE 802.15.4 devices may use either IEEE 64 bit extended addresses or, after an association event, 16-bit addresses that are unique within a PAN. In 802.15.4, the maximum size of the PSDU (Physical layer Service Data Unit) is 127 bytes. With the 25 bytes of the MAC sub-layer (without security), this results in 102 bytes at the link level. By adding data link layer security (AES-CCM-128), only 81 bytes remain available at the IP level. We should also take into account the overhead due to the headers of IPv6 (40 bytes), any extension headers, UDP (8 bytes), or TCP (20 bytes). Finally, the user data is low (33 bytes on UDP and 21 by TCP) and does not allow respecting the IPv6 specifications which impose a minimum MTU of 1280 bytes. The compressibility must respect the rules of the 6LoWPAN standard. The reduced, and the uncompressed fields are systematically preceded by encoding bytes to identify the protocol header and its compressible fields. The encoding bytes are generally one byte. The encoding format takes advantage of the fields that are implicitly known to all nodes in the network or can be deduced from the MAC layer. RFC 49443 defines the compression mechanism for IPv6 headers for LowPAN: LOWPAN_HC1. It also includes compression of the UDP header to 4 bytes but does not allow compression of the Checksum, authorized only when upper layer use the tunneling (as IPsec Encapsulating Security Payload tunnel mode). In addition, it restricts the range of UDP ports from 61,616 to 61,631 to compress this value to 4 bits. This IPv6 header compression can only be applied to local link addresses. To overcome this problem, an IETF draft LOWPAN_HC1g has been published. LOWPAN_HC1g applies to global addresses for IP multi-hop communications. These two compression mechanisms (LOWPAN_HC1 and LOWPAN_HC1g) are complementary. It is, therefore, necessary to implement both of them. Today, the 6LoWPAN group proposes to use LOWPAN_IPHC. It allows to replace LOWPAN_HC1 and LOWPAN_HC1g. IPHC bytes result from compression of the IPv6 header. They mainly integrate quality of service information, next headers, the number of hops, and the source/destination addresses compressed. With LOWPAN_IPHC the compression rate depends on the type of communication:

- For communications over a local link, the IPv6 header can be reduced to 2 bytes (1-byte Dispatch and 1-byte LOWPAN_IPHC);
- For communications requiring multiple IP hops, the header can be compressed to 7 bytes (1-byte Dispatch, 1-byte LOWPAN_IPHC, 1-byte Hop Limit, 2-bytes Source Address, and 2-bytes Destination Address).

6LoWPAN does not concern only the IPv6 header, but also concerns the UDP header. However, UDP does not dispose of a next header field, that is why it was necessary to compress the header of the upper-layer protocol. This 6LoWPAN compression variant is named 6LoWPAN-GHC [31].

### 3.4. HIP DEX Packet’s Format Analysis and Proposed Compression

**Figure 2:**
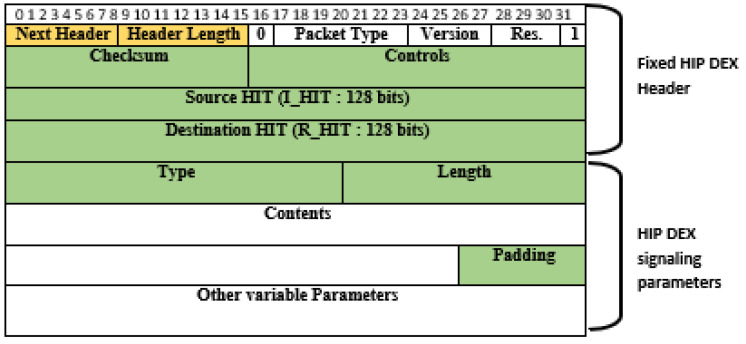
Fixed HIP DEX packets header [9].

In this section, we present the proposed compression in our previous work in [9], then we explain the possible optimization in compression with the LC-DEX solution, comparing with [9]. We distinguish three types of packet content: (a) static information that can be omitted, (b) redundant information that can be compressed and (c) information that is not compressible or should not be compressed. All HIP packets have a fixed header of 40 bytes. As presented in Figure 2, each packet is composed of a fixed protocol header and a varying number of parameters. These parameters reach the actual protocol information. Fixed header and HIP parameters both contain information that is useful in the context of Internet-based communication, but that is unnecessary during packet transferal in constrained network environments.

#### 3.4.1. HIP Header Fix Parameters Compressibility

In HIP DEX as such in HIPv2, there are eight basic HIP packets. The mandatory ones for the handshake process are (I1, R1, I2, R2). Details on these messages were presented in Section 3.2. The rest of the messages are used for signalization: UPDATE, NOTIFY, CLOSE and, CLOSE_ACK. The fixed HIP header is similar in design to the IPv6 extension header. It starts with the required Next Header and Header Length fields and contains an 8-byte alignment for the HIP DEX packet content. This content is constant for all HIP packets and can be concluded from lower layers by summing the sizes of the frames (e.g., MAC service data units) referring to the same HIP packet, with the elimination of the excluded 8 bytes cited above. Consequently, the header length byte can be compressed. As such, the specific part of the HIP header begins with information that is necessary for the correct analysis of a packet, that is, the packet type and the protocol version. As there are eight packet types in HIP DEX, five bits are sufficient for coding the packet type field rather than seven bits according to the proposition in [32]. In our solution, we proposed to remove both NOTIFY and CLOSE_ACK messages. So, the total number of exchanged HIP messages becomes six, and only ***three bits are sufficient for coding the packet type field***. The HIP Version field (VER.) is four bits, and there was not defined version for HIP DEX separately from HIP BEX. So, currently, the existing version is 2 for HIP BEX and consequently the same for DEX. There are two possibilities: (1) the two communicating sensor nodes use the same version; (2) different versions are executed in the HIP peers and if it is the case we may assume that the 6LoWPAN bridge router (6BR) is charged by the adaptation for the incoming packets from the Internet. In our work, we suppose that the communicating peers use the same version. The *version field* maybe, as a consequence, compressed for the generating packets from the sensor nodes and carried unchanged for those coming from outside the network. Additionally, the following three bits are *reserved (RES.)* for future use and set to zero, that is why they can be eliminated. Thus, the checksum is covering the source and destination addresses in the IP header, it MUST be recomputed on HIP-aware NAT devices. So, the 6LoWPAN packets sustaining HIP packets will be fragmented in the 6LoWPAN layer and specifically in the 6BR. So, keeping the original checksum value will be useless. Consequently, we can remove the *checksum* field from the HIP header in the WSN board. R1 and I2 packets contain one bit (A-bit) that is set when the Responder’s or Initiator’s HIT, is anonymous, respectively, and if it should be stored or not by the peer. The management of anonymous nodes must be removed in the context of constrained networks such as an IoT based-WSNs. *A-bit* is a part of the controls bit array (16 bits) that we, accordingly, revoke safely. In the same context, *HIT_R* as redundant information can even be completely revoked in both R1 and I2 messages which reach the source host identifier information. The two *fixed bits* 0 and 1 in the header are used for compatibility issues and must be set only in implementations referring to particular specifications and so, they can be eliminated. Another important target for compression in the header is the HIT_I field that has the heavy length of the header (128 bits). As we proposed to revoke the HIT_R [9], we focus at this stage only on the HIT_R. HIT is a precision representation of the Host Identifier and that is similar in structure to IPv6 addresses. HITs are composed of three components: (a) an IANA fixed 28-bits prefix used to differentiate HITs from standard IPv6 addresses; (b) a code identifier to indicate the compression algorithm used to generate HIT from HI (4 bits); and (c) left-truncation of the HI to 96 bits as a representation of HIT from HI (compression of HI). According to [19], *HIT* fields can be reduced to 96 bits, due to the omission of the HIT prefix (the first 32 bits) and maintaining only the suitable generated value. We propose to minimize the *packet type* field from seven bits to five bits, as proposed by the authors of [32]. As the packet type field cannot exceed the actual standardization of HIP DEX protocol because there are eight types of HIP packets, where the maximal value is 19 (the packet type values in I1, R1, I2, and R2 messages are respectively 1, 2, 3, and 4. With UPDATE, NOTIFY, CLOSE, and CLOSE_ACK, the packet type field takes the values 16, 17, 18, and 19 respectively). The performed amelioration in the proposed compression in this paper and obtained results are presented in Table 2.

#### 3.4.2. HIP DEX-6LoWPAN Header’s Compressibility

**Figure 3:**
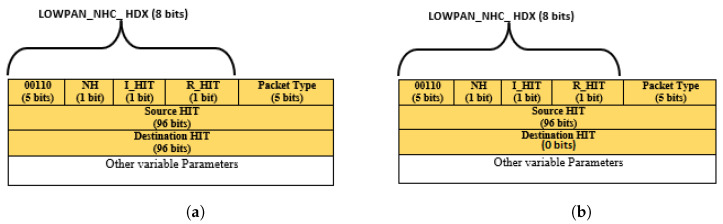
Proposed Compressed 6LoWPAN HIP DEX Header (in yellow). (**a**): Performed results in the conference paper [9]. (**b**): Results obtained with LC-DEX.

**Figure 4:**
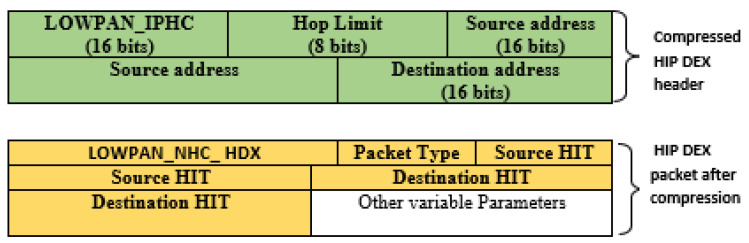
Resulting compressed IPv6/HIP DEX packet.

To apply a 6LoWPAN header compression in a given protocol, it is required to modify the existing NHC encoding or determine a different NHC for the target protocol with different ID bits. The first proposition enforces modification in the current standard. We judge this solution as not a kind one. The possible second solution, used in this paper, is to propose an extension to the 6LoWPAN standard such as the approach applicable to distinguish NHC from GHC. Among encoding bits in the IPHC, is the NH bit that, if set, indicates the next header is compressed using NHC. The NHC is used to encode the IPv6 extension headers and the encapsulated header protocol. Generally, the NHC encoding is only one octet and contains variable-length ID bits and the encoding bits for a specific header. Compression possibilities for different next headers are concerned a bit motif that is variable fixed immediately after the LOWPAN_IPHC compressed header. The next header compression format must be defined after determining obligatory by the perceived frequency of using that format. As HIP DEX header will be associated with the 6LoWPAN header after compression. We can consider that the HIP header is the adaptation of the 6LoWPAN extension header. Therefore, we propose a 6LoWPAN Next Header Compression for HIP DEX header (LOWPAN_NHC_HDX). The fixed HIP DEX packets header is presented in Figure 2. The suggested encoding bytes of the compressed HIP DEX header is defined in Figure 3. The resulting compressed IPv6/HIP DEX packet is presented in Figure 4.

## 4. The Proposed Optimization in HIP DEX Communication Energy Consumption

**Figure 5:**
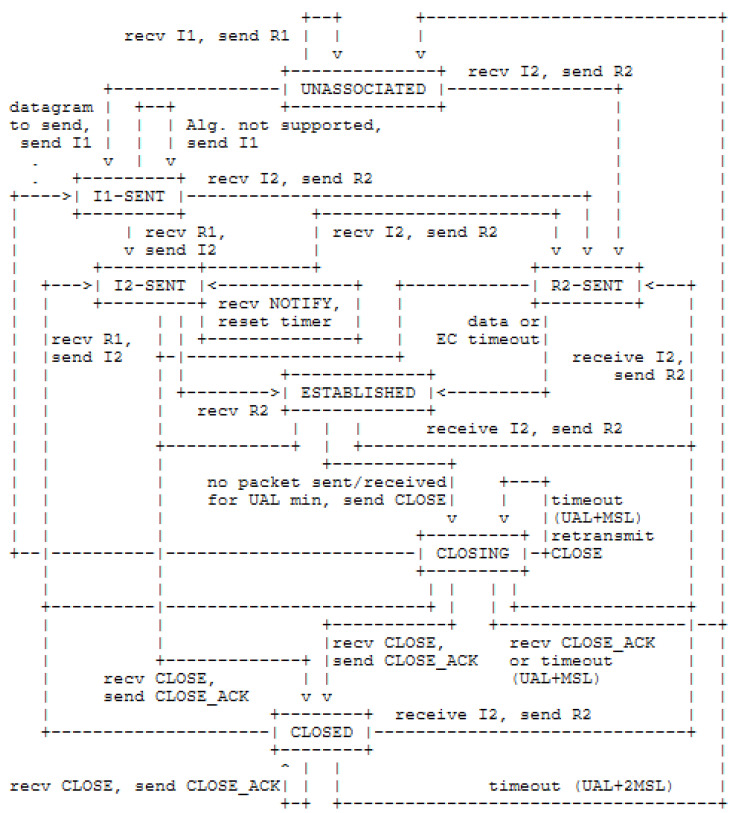
HIP state machine diagram [5].

Over a communication in a multi-hop architecture, HIP is transported over Layer 2 only on the first hop, then routing packets are carried by the 6LoWPAN. For the best optimization and reduction of energy costs of IoT end-devices, we are interested in the four signaling messages UPDATE, NOTIFY, CLOSE, and CLOSE_ACK. These messages are exchanged after the success of the establishment of the HIP association. Among these messages, the NOTIFY is OPTIONAL, but the remaining three HIP packets are mandatory. The reception of errors (e.g., of notification information) MUST be considered only as informational, and there is not any modification of the HIP state machine purely based on the received NOTIFY message. We propose to revoke NOTIFY packet as a consequence to optimize the energy consumption of the HIP DEX handshake process besides our proposal of the HIP header compression discussed in Section 3.4. Our proposition of the removal of NOTIFY packet is the argument, also, by security consideration. Hence, the responder uses optional transmission of NOTIFY messages to inform the initiator by the reception of an I2 message. However, the responder can not guarantee the authenticity of this packet as it did not yet set up the Master Key Security Association [16]. So, an attacker can send spoofed reception acknowledgment for an I2 packet and signal an arbitrary I2 processing time to the initiator. For example, indicating lower processing time for I2 messages induces a premature transmission by the responder. Another possible optimization is concerning the CLOSE and CLOSE_ACK messages. The CLOSE message initiates the termination procedure, and the CLOSE_ACK is sent to confirm the termination of the process. When the initiator sends the CLOSE message, it waits for a waiting period until receiving the CLOSE_ACK message. This waiting time is defined as the period that waits for the device if no packet is sent or received (UAL: Unused Association Lifetime). UAL is important for a constrained IoT node because it can not, mainly, be switched to an energy-saving state. Therefore, we have proposed to remove the confirmation message. The authors of [22], also proposed to remove CLOSE_ACK messages (in HIP DEX protocol), but their solution is feasible only if the responder is a powerful network device with high capabilities in terms of memory and computation (They implemented the responder as a laptop computer). As a replacement scheme, we propose to terminate, immediately, the connection, by the initiator, when the CLOSE message is sent to the CTTP (waiting time for the initiator is null). The CTTP is charged to forward the termination message to the responder. We treated the case where the CLOSE message is lost during the transmission. In this situation, the initiator terminates the connection, but it is not the case for the responder that normally waits for a backup time to terminate the connection or retransmission of the lost message. The maximum required time for a TCP segment to be spent in the network is called the MSL (Maximum Segment Lifetime). The standard HIP DEX state machine is presented in Figure 5.

Since it is not feasible for IoT devices (like is the case in [22]), we assume that the CTTP (see Section 4.1, for more details), sends a repeatable CLOSE message for the responder, immediately after receiving the CLOSE message from the initiator. We consider the following parameters:

- MSL_lp: The MSL time required to the last packet sent by the sender’s HIP to attend the receiver’s HIP successfully;
- $MSL\_cttp=\frac{MSL}{2}$: The time spent by the CLOSE packet sent by the initiator to attend the CTTP. We consider that it is the same interval time spent by the CTTP to send the CLOSE packet to the responder;
- UAL_r: The UAL waiting time of the responder for the reception of a message, such that UAL_r<UAL. If UAL_r=UAL, the responder may tear down the HIP association;
- P_close: The time interval that the CTTP does not exceed to send repeated CLOSE messages to the responder.

**Figure 6:**
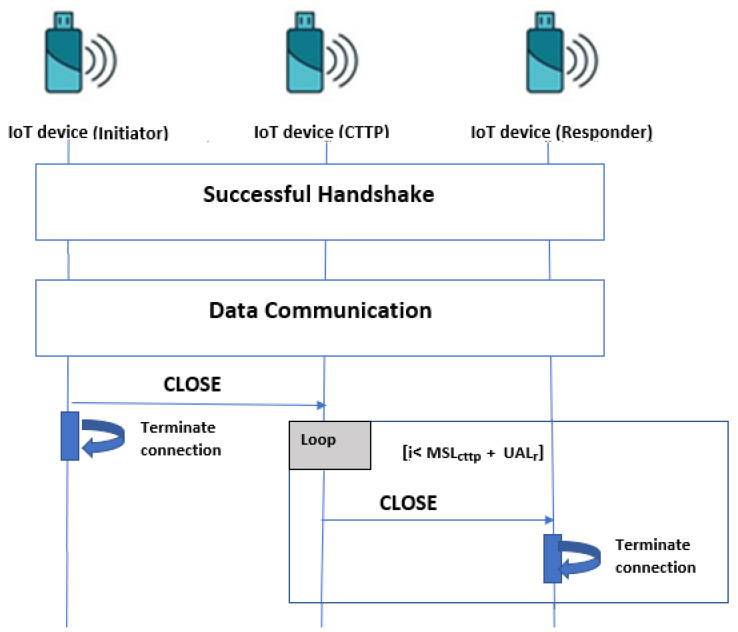
Proposed HIP association termination.

The CTTP iterates in sending the CLOSE messages to the responder during a maximum period P_close as P_close<MSL_cttp+UAL_r. In the HIP standard, the responder waits for (MSL)× 2 + UAL period (in minutes) before tearing down the active association even if no CLOSE packet was received. At the worst case, in LC-DEX, the responder is waiting for MSL_lp+(MSL_cttp)+P_close and at the best case, the responder waits only for MSL_lp+(MSL_cttp)× 2. Within this solution, the responder terminates, certainly, the connection with a minimized waiting period. We can assume that we minimize until zero the probability of losing the CLOSE message. If a CLOSE message reaches the responder when the connection is already terminated (because CTTP still iterates sending CLOSE packets), it has no effect. Figure 6 describes the proposed HIP DEX connection termination. The minimization of the number of HIP DEX messages (**from eight messages to six**) reduces the computation resources for their processing by the device and also, some bandwidth of the communication channel. Furthermore, the benefits of the proposed association termination solution are that it is suitable for constrained devices in IoT with lower capabilities and is applicable in both HIP DEX and HIP BEX.

### 4.1. HIP DEX Distribution Scheme

Multi-hop routing and processing in constrained networks such as WSN can lead to transmission latency delay not tolerated, even with higher levels. In the following, we propose a distribution model so that we can minimize the communication and computational overheads. The HIP DEX computational key agreement is known for its lightness comparing with the original protocol version (HIP BEX) and it is the reason that HIP DEX was designed. The proposed distribution model introduces a Collaborative Trusted Third Party (CTTP) charged to alleviate the HIP-peers by executing the heavy cryptographic primitives of the HIP DEX handshake in WSNs. To achieve that, the HIP DEX peers delegate the most consuming CPU tasks to the HIP third party. The CTTP, in our scheme, is selected among the internal IoT domain’s 6BR, except the edge router. The CTTP is, also, charged with the compression and decompression of HIP DEX packets’ to minimize the computation overheads of HIP-peers. The following hypotheses are supposed to implement our solution:

- Constrained nodes can achieve symmetric encryption;
- CTTP can perform asymmetric encryption because it is the most energy-consuming operation in the HIP DEX process;
- Each node must have a direct connection to the selected CTTP nodes during the initialization phase;

The distribution model is defined by two principles phases: The initialization phase and the establishment phase. Concerning the second phase, we propose a new technique that alleviate considerably the energy consumption of the HIP peers during association establishment. Section 4.1.1 and Section 4.1.2 detail these two steps.

#### 4.1.1. Initialization Phase

Only the IoT HIP domain network is concerned with the steps of this phase. The outsider Internet hosts are not conscious of that. This phase initiates all the security parameters for all the communicating entities. In general, there are two methods to distribute the generated public key: manual and automated.

- Manual method: This solution requires a distribution of the public key to the end devices by uploading it to their memory manually. So, there are no messages exchange to distribute the key. The pair-keys must be changed periodically and charged again to all end devices to resist key recovery. The manual distribution key is beneficial from a security point of view because attackers can not divine the public key. However, it is not practical for IoT networks qualified by their wide geographical areas;
- Automated method: Despite the energy efficiency of the manual key distribution method (omitting the key transmission), it is not practical for many IoT applications where the administrator must charge the key in devices memories at least once a month.

We are adopting the automated method in our solution, which is more efficient than the manual method in the context of IoT and does not require any administrator intervention. This method considers the key to be exchanged in the HIP messages during initial communication. Alternatively, it is stored in the device memory and is valid even after the HIP association is terminated.

#### 4.1.2. New HIP DEX Secure Lightweight Association Establishment Phase

**Figure 7:**
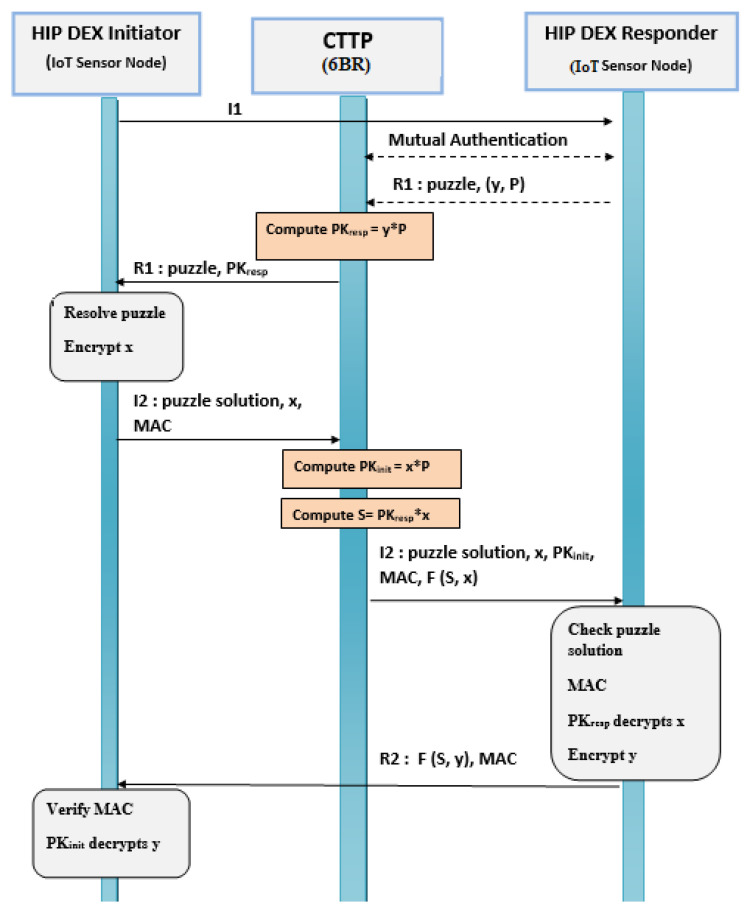
Proposed HIP DEX Secure Lightweight Association Establishment Phase.

On the issue of the Secure Lightweight Association (SLA) establishment phase, the peers establish the HIP association for exchanging data over a secure communication channel. The HIP DEX SA may be concluded in two steps. Firstly, the initiator and the responder agree together publicly on an Elliptic Curve. Then, both the Initiator and the Responder choose random values *x* and *y*, respectively, and also publicly choose a point P from the curve. These two exchanges do not need any secure channel between the initiator and the responder. The most expensive computational part of all elliptic curve cryptosystems is the scalar multiplication in the form of $k_{}$ × $P_{}$, where *k* is an integer and *P* is a point, from the curve, called the generator. For this reason, we propose to delegate all scalar multiplication operations to the collaborator node (6BR in our solution). By definition, the node initiating a HIP DEX is the Initiator, and the peer is the Responder. This distinction is negligent once the handshake is taken place and the communication is operational. We propose, in this paper, a modification of the standard’s HIP DEX SA to alleviate the overhead of scalar multiplication in the HIP peers during the computation of the Host Identities (HI). To our knowledge, we are the first to propose such a solution in HIP DEX and with a minimal number of collaborative third parties (only one). The modification of the proposed SA is shown in Figure 7. The new proposed authentication process is as follows:

1. The process starts with sending a trigger packet, I1, by the initiator to the responder;
2. After receiving I1 and a successful mutual authentication with the CTTP, the responder sends the R1 packet containing the puzzle and (*y*,*P*) parameters to the CTTP;
3. The CTTP performs the scalar multiplication operation $PK_{resp}$ = $y_{}$ × $P_{}$ where the resulting $PK_{resp}$ is the responder’s public key (HI). Then, the CTTP forwards R1 packet to the responder;
4. The initiator sends the puzzle solution, the random value *x*, and MAC in the I2 packet to the CTTP;
5. The CTTP computes $PK_{init}$ = $x_{}$ × $P_{}$ that represents the initiator’s public key (HI) such that the CTTP stores the generator point P received in step 2. The CTTP, also, computes the secret’s initiator as S = $PK_{resp}$ × $x_{}$. Then, CTTP forwards the received I2 packet, including the computed S, to the responder;
6. On receiving I2, the responder checks the puzzle solution and does not need to calculate the secret key because it has already been obtained from the CTTP. In HIP DEX the secret key calculated by the initiator and the responder are the same;
7. The fourth message R2, also, has the MAC value, the key wrap parameters F(S,y) and it finalizes the handshake.

In this paper, we propose a modification of the SA phase so that we minimize the number of exchanged messages between the collaborator and the HIP-peers that deal with the minimization of the communication costs. Besides, we detail in this work the mutual authentication process between the collaborator (CTTP) and the HIP-peers (originally only with the responder node).

##### Mutual Authentication Responder/CTTP

To perform an efficient and light mutual authentication between the CTTP and the responder, we propose to implement a One Time Password (OTP) authentication process regarding the lower capabilities of both the HIP-peers. Likewise, the solution given by authors in [33] regarding the Micro–Macro IoT Authentication Paradigm, we propose an authentication scheme based-OTP between two sensor nodes as the followings:

1. The adopted communication mechanism is the Direct Sensor Access (DSA) between the CTTP and the HIP-peer (Responder);
2. The first step is about the reception of the hashed password, that will be used as an ID by all the sensor devices in the HIP IoT domain. Indeed, the base station is responsible for the generation of the OTP IDs that are updated after a certain period and calculated by a dedicated algorithm. These OTP IDs are stored in a local database in the target base station;
3. The second step is about the verification if it is the first time to communicate with the CTTP. If it is the case, the mutual authentication using the OTP ID received, previously, from the base station is used to identify each other. Otherwise, there is no need to perform mutual authentication, and R1 is sent to the CTTP.

To verify the knowledge of the CTTP as identified previously, we propose to memorize the IP address of the CTTP by the HIP-peers after the first successful mutual authentication. A new mutual authentication is performed when one of the following cases arises: (1) When the IoT devices receive the new OTP-ID; (2) If the network connection between the HIP-peers and the CTTP is discarded.

The proposed SLA scheme has another advantage. Besides the energy efficiency, the security threats are restricted because we consider that the HIP-peers and the collaborator nodes are in the same IoT domain.

## 5. Introduction of a Domain Name System in a HIP DEX Opportunistic Mode

In HIP DEX, if the initiator does not know the HIT_R, it can initiate the HIP handshake with the I1 packet where all bits of HIT_R are set to zero. This connection mode is called opportunistic. In Section 3.4, we detailed the arguments to revoke HIT_R and to minimize HIT_I size. We can optimize the communication overhead of HIP DEX packets by alleviating their size in omitting both HIT_I and HIT_R with the introduction of a Domain Name System (DNS) in an HIP DEX opportunistic mode. The proposed scenario implements the CTTP as an HIP DNS server and steps for generating and distributing HIT from HI are described in the following:

1. After computing the $PK_{resp}$, the responder’s HI (HI_R): the public key (see Figure 7, Section 4.1.2), the CTTP computes the HIT_R from HI_R using FOLD function to get the 96 bits-compressed format of HI_R and then stores a binding of HI_R and HIT_R corresponding to the responder in its local memory;
2. The CTTP sends the calculated HIT_R to the initiator;
3. After computing the $PK_{init}$ as the initiator’s HI (HI_I) using FOLD function to get the 96 bits-compressed format of HI_I and then stores a binding of HI_I and HIT_I corresponding to the initiator in its local memory;
4. The CTTP sends the calculated HIT_I to the responder.

Introducing a DNS server to distribute HITs for HIP peers has the major advantage of the minimization in the computation cost due to the delegation of the HITs’ computation to the CTTP. This solution encourages the establishment of a secure association in HIP DEX in an opportunistic mode with minimal energy consumption. This solution can be generalized to generate the HIT_SUITE_LIST that represents a group of initiator’s and responder’s IDs. The HIT_SUITE_LIST is defined in HIP DEX to allow the initiator to identify the IDs supported by the responder and the same benefit for the responder. With our proposed solution, both the initiator and the responder receive the HIT of each other from a third party and be sure that it is a legitimate HIP peer. Security threats and mechanisms to secure the third party as it has a main task in the handshake are discussed in Section 8.

## 6. Evaluation and Experimental Results

The fundamentals of cryptographic techniques used in IoT should respect the constraints of sensor nodes and the evaluation criteria are mainly the complexity of code, packets’ sizes, processing time, and energy consumption. In the rest of this paper, we demonstrate that we respect all of these criteria. Our network setup and our evaluations were conducted on FIT IoT-LAB [10] that provides a large-scale infrastructure facility and experimental platform suitable for testing small wireless sensor devices and heterogeneous communicating objects. The FIT IoT-LAB is a real experimental testbed that allows users to implement a real experimental scenario since it embeds many Access Points in the building. It provides full control of network nodes and direct access to the gateways to which nodes are connected, allowing researchers to monitor several network-related metrics. FIT IoT-LAB features over 2000 wireless sensor nodes spread across six different sites in France. For our experimentation, we chose nodes from the site of Grenoble. The IoT-LAB hardware infrastructure consists of a set of IoT-LAB nodes. A global networking backbone provides power and connectivity to all IoT-LAB nodes and guarantees the out-of-band signaling network needed for power and command purposes and monitoring feedback. Various hardware platforms are available on FIT IoT-LAB: WSN430 Node, M3 Node, A8 Node, B-L072Z-LRWAN1 LoRa kit, and so forth. We have executed our experimentations on A8 Nodes because they allow running a high-level OS like Linux and Contiki. Another central reason for this hardware choice is that our code implementation of the HIP DEX protocol is written in C++ and executed with the Makefile method for Linux operating system. It is our implementation described in our previous work in [9] where we added necessary libraries for the compression (explanation is below in this section). We have modified the code so that it enables handshakes in 6LoWPAN architectures. A8 node’s components are described in Table 3. Protocols used at the different network layers are detailed in Table 4. We evaluated the handshake delays explained in the following sections, in a WSN based-IPv4-IoT, and a WSN based-6LoWPAN-IoT architecture. In the second one, we configured three A8-M3 nodes: the first node as Initiator, the second node as Responder, and the third one as 6LoWPAN bridge router (CTTP). Both the initiator and the responder are configured as CoAP servers.

### 6.1. Network Model

**Figure 8:**
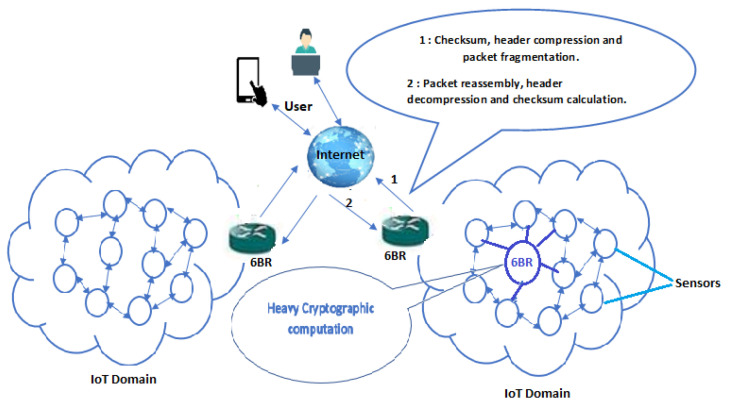
Adopted 6LoWPAN-based IoT architeture.

Figure 8 shows the adopted architecture for the evaluation of the efficiency of transmission delays of HIP DEX handshake before and after the proposed compression.

### 6.2. Minimization of HIP DEX Handshake Packets’ Sizes

In this section, we present the performance of the HIP DEX packet header compression discussed in Section 3.4. In Table 5, we present the handshake packets’ sizes before and after the compression. We deduce that our novel proposed compression scheme in LC-DEX achieves reduction gains of all HIP DEX packets sizes’ about *2 times* comparing with results obtained in our previous work in the conference paper [9]. For this aim, we give a comparison of the amelioration in packets’ sizes performed by our work in [9] in comparison with standard HIP DEX and obtained results of LC-DEX comparing with standard HIP DEX.

These reductions have a direct consequence in minimizing the computational and communication overhead of the overall HIP DEX handshake. Our solution gives better results than other existing solutions (see Table 6). We compare our results with HIP-based solutions and other solutions based on Extensible Authentication Protocol (EAP) and Authentication and Key Agreement (AKA) protocols in the context of IoT. LC-DEX performs an excellent reduction in total exchanged bytes during the handshake that amount to 90% comparing with PANA/EAP-TLS. Comparing with D-HIP, LC-DEX need the half of exchanged bytes during the handshake. An 80% of reduction is performed in comparison with D-HIP.

### 6.3. End-to-End Transmission Delay Reduction

In this section, we present the experimental results of the handshake regarding the improvement of the total E2E transmission delay after the compression of HIP DEX packets. For doing so, we evaluated all delays involved in the transmission delays of the packets’ handshake regarding the transmission delay of the handshake process between the Initiator and the Responder, the propagation, the processing, and the queuing delays. A granular comparison with previous works that evaluate the same delays composing E2E transmission delays are presented in the following sections.

#### 6.3.1. End-to-End Transmission Delay

Reduction of transmission delays of our experiments is done under a real mesh WSN-based IoT network. We conducted different evaluations in our previous work [9] regarding the transmission delay of HIP DEX handshake. Experiments were done in a WSN based-IPv4-IoT architecture (a) and a WSN based-6LoWPAN-IoT architecture (b). The following formula is used to calculate the transmission delay:

$$T_{r}=\frac{L}{R},$$

where:

- $T_{r}$ is the transmission delay in seconds;
- L is packet length in bits;
- R is the transmission rate in bits/s. In our experiment, we implement a Constant Bit Rate (CBR) model equal to 250 kb/s.

**Figure 9:**
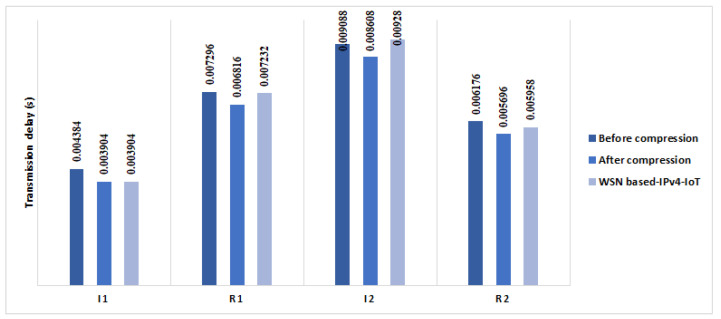
Transmission delays results before and after the proposed HIP DEX header’s compression comparing with transmission delays in an IPv4 Mesh_under architecture [9].

We observed that the overall transmission delays of handshake packets in (a) are about 0.026 s (see Figure 9). In (b), we evaluated the transmission delay in a kindly real IoT network where we implemented the 6LoWPAN, CoAP, and RPL protocols. The transmission delays are calculated in two situations: standard HIP DEX handshake packets and compressed HIP DEX handshake packets. The overall transmission delay during the handshake is about 0.029 s of standard HIP DEX handshake and 0.027 s of HIP DEX handshake after compression (see Figure 9).

**Figure 10:**
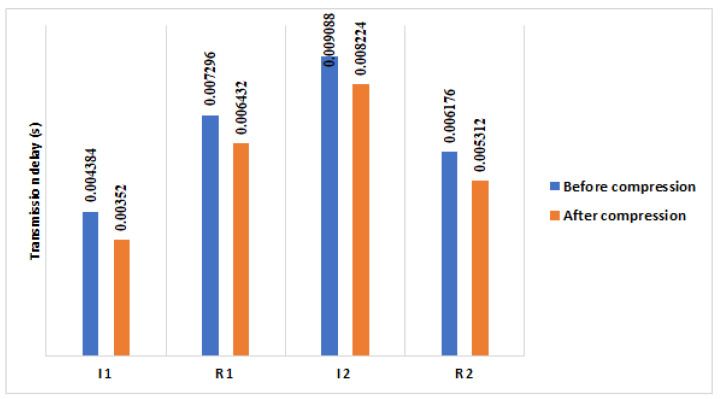
Transmission delays results before and after the proposed HIP DEX header’s compression in LC-DEX solution.

We performed a transmission delay ratio gain of 7%. At the same conditions and experiments performed in [9] to evaluate the transmission delays of HIP DEX handshake in the WSN based-6LoWPAN-IoT, we measured the transmission delay after the improvement of the HIP DEX packets’ sizes presented in Section 3.4. Obtained results are presented in Figure 10. The total transmission delay of the exchanged packets during the handshake in LC-DEX is about 0.023 s. We can observe that LC-DEX performs a percentage gain of about 8% comparing with results obtained previously in our conference paper (see Table 7).

We also evaluated our results (LC-DEX) in comparison with our previous work in [9] and with the recent work, P-HIP [36], which studied the transmission delay during the handshake. LC-DEX improved an excellent percentage gain of about 97.82% in comparison with P-HIP and about 6% in comparison with our work presented in the conference paper [9], (see Table 8).

#### 6.3.2. Propagation Delay

For calculating the propagation delay of HIP DEX handshake packets, we use the following formula:

$$P_{r}=\frac{D}{T_{s}},$$

where:

- $P_{r}$ is the propagation delay in m/s;
- D is the distance between the Initiator and the Responder. In the experiment, we chose nodes with 1 meter of distance;
- $T_{s}$ is the propagation speed, which is calculated as speed of light×velocityfactor. The speed of light is 2.998 × $10^{8}$ m/s and the velocity factor = 1 because the propagation medium is the air.

The $P_{r}$ is equal to 0.003 µs for one packet. Consequently, $P_{r}$(handshake) = 0.012 µs. It is a negligent value that has no impact on the total handshake transmission delay.

#### 6.3.3. Processing Overhead

The processing delay ($P_{\mathrm{ps}}$) is defined as the elapsed time by a network device (generally the router) to process the header of a packet. Because the processing time is dependent on the header length, compressing the header packet (from 40 bytes to 25 bytes in the maximum case) has a direct impact in minimizing the overall E2E transmission delay of the handshake. In our evaluation, we measured the $P_{\mathrm{ps}}$ belonging to the HIP DEX handshake in two different architectures: the first architecture (a1) is a WSN based-IPv4-IoT that represents a kind Peer-to-Peer Wireless Sensor Network where the communication between the Initiator and the Responder is established with no need for an intermediate router and, the second architecture (a2) is the WSN based-6LoWPAN-IoT. For measuring the $P_{\mathrm{ps}}$, we get results under Linux installed on A8-M3 nodes using *top* command on the Initiator and the Responder. We performed 100 handshakes, and we noted the processing delays in the Initiator and Responder sides. Note that $P_{\mathrm{ps}}$(I1,I2) is processing delay of both I1 and I2 combined and the same for $P_{\mathrm{ps}}$(R1,R2). The obtained results in (a1), show that $P_{\mathrm{ps}}$(I1,I2) is about 0.083 s and, $P_{\mathrm{ps}}$(R1,R2) is about 0.078 s. In (a2) and before compression of HIP DEX packets, $P_{\mathrm{ps}}$(I1,I2) = 0.094 s and, $P_{\mathrm{ps}}$(R1,R2) = 0.084 s. After compression, $P_{\mathrm{ps}}$(I1,I2) = 0.078 s for both I1 and I2 packets and $P_{\mathrm{ps}}$(R1,R2) = 0.078 s. We compared the obtained results in LC-DEX with our previous results in [9].

**Figure 11:**
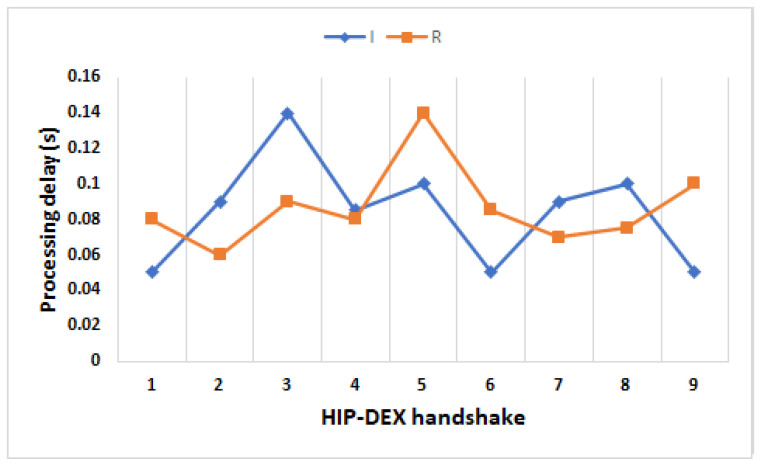
Processing delays on the initiator and responder sides.

Table 9 recapitulates the obtained results from [9] in (a1), (a2) before compression and (a2) after compression. In (a2) after compression and in comparison with (a1), we perform a processing handshake that amounts to 6% for I1 and I2 packets. In (a2), we deduce that the gain in a processing delay of the HIP DEX handshake amounts to 44 ms (12.5%) with the proposed compression, in comparison with the standard HIP DEX. Table 10 presents a comparison of the HIP DEX processing packets in the initiator and the responder sides with HIP previous works. Figure 11 presents an overview of the consumed time during the processing of initiator’s and responder’s daemon. We can observe that the maximum value is 0.146 s, and it is the same for processing initiators’ packets and responders’ packets.

For the comparison with previous works, we considered the maximum value of the processing delays (see Figure 11) for both the initiator and the responder. The performed percentage gain is better, if we consider the average values. Comparing with [34], LC-DEX performs delay’s gain of 85% and 82% in the Initiator and Responder sides, respectively. Comparing with [35], we performed 92% and 80% of delay gains in the Initiator and Responder side, respectively. LC-DEX gives 92% of delays gain for both Initiator and Responder sides. LC-DEX is faster, more than two times faster than P-HIP. In comparison with our solution in [9], the LC-DEX delay gains amount to 6.41% for both Initiator and Responder sides.

#### 6.3.4. Queuing Delay

The Queuing Delay ($T_{q}$) depends on the level of network’s congestion. It is the time that a packet waits in the queue until it gets processed. The average of ($T_{q}$) is calculated as follows:

$$T_{q}=\frac{(\mathrm{PN}-1)\times L}{2\times R},$$

where:

- $T_{q}$ is in seconds;
- PN is a number of packets to process;
- L is the packet length;
- R is the transmission rate in b/s. As indicated in Section 6.3.1, R = 250 kb/s.

As we have four packets in the handshake, we are considering L as the average of packets’ lengths. The average handshake packets’ lengths in WSN based-IPv4-IoT architecture is 166 bytes. Obtained $T_{q}$ value is 0.007 s. For calculation of the $T_{q}$ in the WSN based-6LoWPAN-IoT architecture, we measured $T_{q}$ after the proposed compression. The average of the 4 packets’ lengths is equal to 226 bytes before compression and 211 bytes after the compression. $T_{q}$ has the same value before and after the compression, about 0.01 s.

#### 6.3.5. Overall E2E IoT Handshake Time Reduction

For calculating the total E2E handshake time of one handshake packet, we use the following formula:

$$E2E\;\mathrm{transmission}\;\mathrm{delay}=T_{r}+T_{p}+T_{q}+T_{\mathrm{ps}},$$

where:

- $T_{r}$ is the transmission delay;
- Pr is the propagation delay;
- $T_{q}$ is the queuing delay;
- $T_{\mathrm{ps}}$ is the processing delay.

Table 11 summarizes the total transmission delays of the handshake obtained during the experiments.

For a further evaluation of the transmission delay performance during the HIP DEX handshake in the protocol stack, we compare the obtained results, between an IPv4 IoT over HTTP domain and a 6LoWPAN over CoAP architecture. We observed, on one side, that the reduction of the overall HIP DEX handshake E2E delay is improved when it is executed in a constrained environment where the protocol stack implements protocols standardized especially for constrained and lossy networks. As we implemented 6LoWPAN, CoAP, and RPL protocols on our network experiment, we deduce that the E2E HIP DEX handshake is minimized by about 32.75% in the WSN based-6LoWPAN-IoT architecture (before compression) and about 41.37% (after compression), in comparison with WSN based-IPv4-IoT architecture (Results from [9]). On another side, the compression of HIP DEX handshake packets ameliorates the overall E2E delay of the handshake by 12.82% (Results from [9]). Table 12 presents a comparison about the overall E2E LC-DEX’s handshake delays with previous works.

### 6.4. Energy Model

We focus our evaluation on the energy consumption during the HIP DEX handshake execution. This evaluation is focused on computation and wireless communications over the whole authentication process. Energy costs regarding cryptographic operations are calculated based on transmission, reception, and listening. Several works do not consider the listening period on their evaluations. Nevertheless, we consider the listening delay has an important incidence on the total energy cost and should not be deduced from the energy cost calculation. Besides, each experiment test iterates 100 times to calculate the average energy consumption and computing latency. The measurements of energy consumption are accomplished by the knowledge of the transmission and listening parameters current in milliAmpere (mA) and time in millisecond (ms) [37]. For calculating the listening duration that takes one packet to be sent from one HIP peer to another, we assume that the collaborator 6BR is one-hop neighboring the HIP-peers. We deduce that the listening time is considered as the latency time of a packet to be transmitted from one node to another, adding the elapsed delay to get the response. This important delay has an impact on the energy communication cost and, hence, on the overall handshake energy cost. This measurement are given on Section 6.4.1. At the end of this section, a comparison with previous HIP-based solutions is made regarding the computational and communication computational costs. To calculate the energy consumption in mJ, we use the following formula equation:

(1) Energy (mJ)=Time×Current×Voltage,

where:

- Time is the execution time (ms) for individual cryptographic operation (time elapsed in CPU operations);
- Current is obtained based on experimental results;
- Voltage is the nominal voltage = 4.8 V.

In FIT IoT-LAB, we can add a monitoring profile in the sensor nodes to obtain the real energy consumption (voltage, current, and power) in real-time execution. As a result, we have obtained that the A8 node consumes only 4.8 V (theoretically, it is between 3 V and 6 V [38]). The CPU has a power less than 300 mW [39] and, as known that for calculating the power, we use this formula:

Power=Voltage×Current.

The IoT-LAB A8-M3 as indicated by its name is equipped by the IoT-LAB M3 object. As a consequence, it uses the same features (sensors, actuators, radio chip) as the integrated M3 sensor. So, the current draw for CPU is about 62.5 mA as a maximal value in the ball level for A8-M3 nodes. The embedded M3 co-microcontroller consumes 42 mA (16 mA at full power, 14 mA for radio transmission, and 12 mA for radio reception). Therefore, the A8-M3 CPU consumes about 20.5 mA as a maximum value. So, the current consumption will be less than this value. In our set of experiments, we randomly chose sensor nodes and implement a Constant Bit Rate (CBR) model equal to 250 kb/s.

#### 6.4.1. Evaluation of the Multi-Hop Energy Communication Cost of the Proposed HIP DEX Compression Model

We start our evaluation by calculating the energy gain of the proposed HIP DEX 6LoWPAN compression header. Our experiments are executed under multi-hop architecture (1 hop, 4 hops, and 8 hops). Note that the distance between the HIP-peers and the 6BR (CTTP) is 1 meter. The energy communication cost of one HIP packet, we calculated using the following formula:

(2) HipEnergyComm (mJ)=[(BUSY_TX×14 mA)+(RX_ON×12 mA)]×4.8 V,

where BUSY_TX is the transmission time and the RX_ON is the listening period [40] and is about 0.000208 s. For calculating the transmission time (BUSY_TX), we use the following formula:

(3) $$\mathrm{BUSY}\mathrm{TX}(s)=PacketSize\times (\mathrm{bytes})\times \frac{8}{250\times 1000}.$$

**Figure 12:**
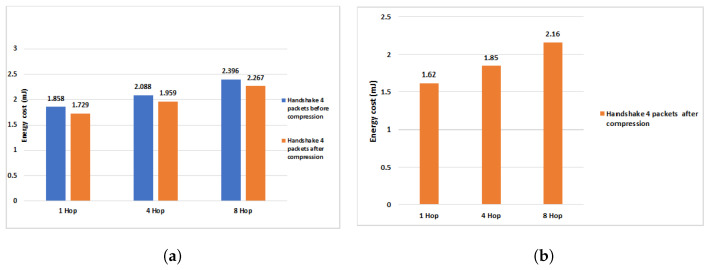
Energy communication costs of HIP DEX handshake (4 packets). (**a**): Calculation done according to results obtained in [9]. (**b**): Results obtained with LC-DEX.

**Figure 13:**
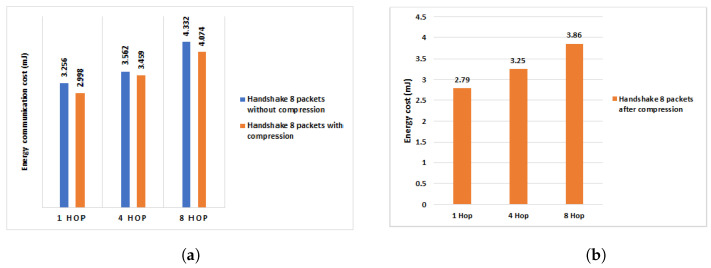
Energy communication costs of HIP DEX handshake packets + signalling packets before optimization (8 packets). (**a**): Calculation done according to results obtained in [9].(**b**): Results obtained with LC-DEX.

**Figure 14:**
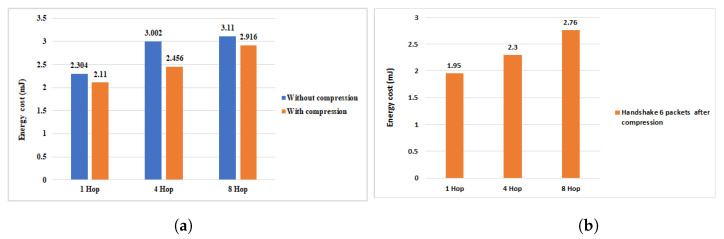
Energy communication costs of HIP DEX handshake packets including the signaling packets (6 packets). (**a**): Calculation done according to results obtained in [9]. (**b**): Results obtained with LC-DEX.

To study in detail the efficiency of our work in terms of energy consumption during the communication of the HIP DEX packets, we conducted the necessary experiments to measure the efficiency of our scheme proposed previously in [9] and the same experiments are conducted with the new compression scheme proposed in this paper. We, firstly, evaluate only the energy consumption when exchanging the handshake packets (4 packets). Then, we execute a full HIP DEX process within the eight corresponding packets (handshake packets + signaling packets = 6 packets). Finally, we evaluate our proposal of optimization discussed in Section 4 (only six exchanged packets: four handshake packets + two signaling packets). Figure 12, Figure 13 and Figure 14 show the obtained results before and after the proposed HIP DEX header was combined with 6LoWPAN compression.

We can observe, from Figure 10, that the percentage gain in the energy communication cost of the four handshake packets on one Hop communication architecture is about 6.95%, 6.2% on four Hops and 5.4% after the proposed HIP DEX header compression on eight Hops.

Regarding the energy communication cost of the handshake including the eight packets from [9] and during the multi-hop transmission architecture, we remark that the compression of HIP DEX header gives an improvement of about 8% on one Hop communication architecture, 3% on four Hops, and about 6% on eight Hops (see Figure 13). As presented in Figure 14, the percentage gain in energy communication cost of the handshake process within six exchanged packets and with the adoption of our solution in [9], after the proposed HIP DEX packets’ number minimization explained in Section 4, is about 8.5% on 1 Hop architecture, 18.2% on four Hop architecture and, 6.3% on eight Hop architecture.

#### 6.4.2. Efficient Reduction on the Energy Communication Costs in Comparison of Our Previous Work

In Table 13, we present the HIP DEX handshake communication results of LC-DEX in comparison to our previous work in [9].

#### 6.4.3. Evaluation of the Computational Cost of the Proposed HIP DEX Compression Model

The computational cost is calculated using the following equation where $T_{cpu}$ is the task execution time in CPU, the current is equal to 20.5 mA and the voltage core is 1.2 V [38]:

(4) $$\mathrm{HipEnergyCompt}=T_{cpu}\times 20.5\;\mathrm{mA}\times 1.2\;V.$$

We can assume that solutions that do not adopt any compression technique as in Ben-Saied et al.’s and D-HIP’s solutions present the most expensive energy consumption in comparison with those that implement compression. Besides, we prove that HIP DEX is a more efficient authentication protocol than HIP BEX in terms of computational cost due to the use of Elliptic Curve Cryptography (ECDH). Our solution minimizes the number of signaling messages in comparison with Ben-Saied et al., C-HIP, D-HIP, Slimfit, and CD-HIP that is the reason for the minimization of the computational overhead and as a consequence the minimization of the overall energy consumption.

#### 6.4.4. Overall Energy Consumption Results Compared with HIP-Based Solutions in IoT

**Figure 15:**
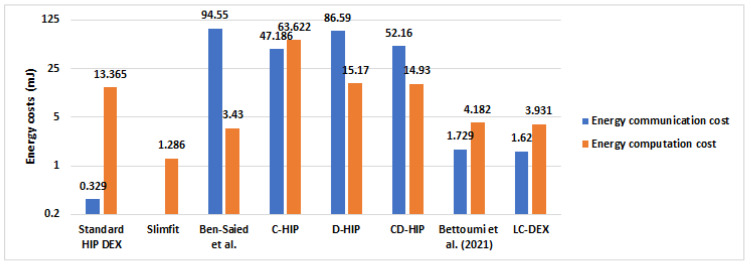
Computational and communication energetic costs of evaluated solutions in comparison with LC-DEX.

**Figure 16:**
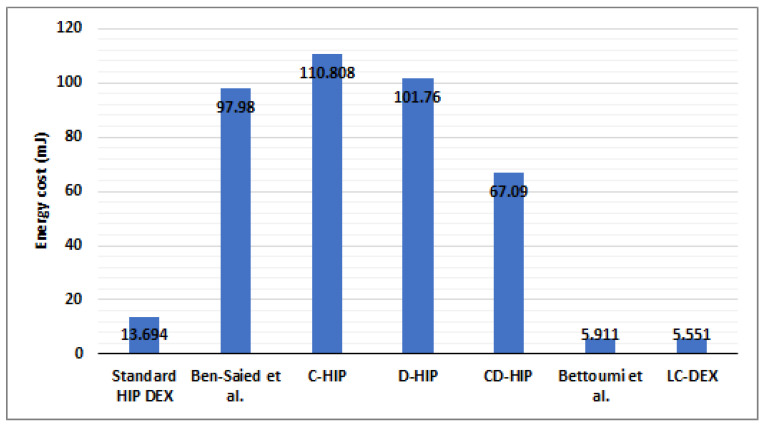
Overall energy consumption.

Energy consumption is a decisive criterion for Wireless Sensor Networks in the context of IoT. In Section 6.2, we present the comparison of HIP DEX packets’ sizes before and after the LC-DEX proposed compression solution of HIP DEX packets. The compression has a direct impact on the computational and communications energy costs. Figure 14 and Figure 15 represent both communication and computational costs of existing HIP solutions. From Figure 15, we remark that solutions that propose compression (C-HIP, CD-HIP, Slimfit, Bettoumi et al. and LC-DEX) reduce considerably the communication overhead. Figure 16 gives a comparison of the overall energy costs of LC-DEX with previous HIP solutions.

Ben-Saied et al., D-HIP, and CD-HIP solutions could be judged as costly energy communication overheads solutions. Ben-Saied et al.’s solution proposes a distribution model based on an important amount of messages involved in the communication. Concerning CD-HIP, the implementation of HIP BEX with its heavy cryptographic primitives and also, the introduction of a third party in the distribution phase without the proposition of a strategy that minimizes the number of exchanged messages between the HIP peers and the third party. CD-HIP has a significant energy consumption that is against the poor capabilities of sensors of IoT networks. Accordingly, the total communication cost is necessary very high in the case of frequent HIP session establishments. In the LC-DEX solution, the combination of the header compression and the omission of two signaling messages (NOTIFY, CLOSE_ACK) of the most efficient bootstrapping candidate for constrained devices (HIP DEX), proves an excellent reduction in energy consumption. The introduction of a collaborator in the distribution model in LC-DEX takes into consideration the minimization of the number of exchanged messages between the collaborator and the HIP peers. In addition, the integration of the third party in LC-DEX has an important role in the alleviation of the scalar multiplication of ECDH cryptographic computation of HIP DEX. Hence, an important impact is the minimization of the HIP energy costs.

### 6.5. Optimization in the Energy Consumption during the Life Cycle of the HIP-Sensor Peers

It is legitimate that when more security is applied, more energy is required. However, it is an absolute necessity that must be adapted with maximum energy consumption optimization. Cryptography computations consume more energy. Within WSN, all involved nodes perform some cryptographic operations. Since WSN is a limited energy and constraint network, some kinds of cryptographic operations are not appropriate. Consequently, it is meaningful to alleviate the heavy cryptographic operations by proposing either a software solution or a hardware solution or both. This kind of intervention should be studied during the sensor life cycle. The life cycle of a sensor can be defined differently according to the application of WSN, such as industrial WSN, medical WSN, and so forth. In this paper, we propose an authentication solution that may be applied in different constrained environments. Thereby, a sensor can be in two main operational states: sleep state and active state. The sleep mode coincides with the lowest energy consumption of a node because it has no interaction with the network. The active state defines two sub-states: idle mode or data transmission and/or reception data packets mode or generation of data. During the authentication process with LC-DEX, we clearly contribute in minimizing the total energy consumption in both communication and computational sides in an IoT network based on HIP process Indeed, computing and generating the keying material are performed during the active sensor state. As we presented in Section 4.1.2, we contributed to lightening the sensor’s charge in the active state. Our new SLA scheme brings two advantages in the sensor energy life cycle, as follows.

- The delegation of the most heavy cryptographic operation of HIP DEX to the CTTP, has a direct impact in minimizing the computational energy cost of the sensor during the active state and more precisely during the generation data mode;
- The exchanged parameters during the transmission of the R1 packet by the responder and during the transmission of the I2 packet by the responder are minimized in the proposed SLA model (see Section 4.1.2, Figure 7). Hence, we contribute to minimizing the energy consumption during the transmission mode for both the initiator and the responder in the active state.

Comparing with standard HIP DEX, LC-DEX improve an energy gain about 60% (see Table 14). Consequently, the minimization in the energy cost of the sensor node during the authentication process has a direct impact in the extension of its life. Studying in details the proportion of the energy consumption caused by the HIP DEX authentication process and the tangible extension of the HIP sensor device are with high importance and we are working on it as a future direction.

## 7. Memory Requirements

To fix the memory size required by our implementation, we measured in real time the memory consumption of our solution by execution *top* and *free* Linux commands. It is worth explaining how we obtained these results. The measurements were conducted in comparison with the memory size occupied initially by the Linux operating system before launching any task. After that, we measured the memory size in the initiator node after executing the corresponding module code. The same steps are performed in the responder node. Then, we subtract the increase in memory after executing the HIP DEX code. The ROM of the A8 node is On-Chip boot memory with programs like X-Loader/U-boot for booting the node. So we will not expect a size variation during the execution of our program. Table 15 presents the amount of data in RAM on both the initiator and the responder sides.

Our implementation of LC-DEX is defined as an executable software under Linux OS. Hence, we focus our research on lightening the authentication process especially for both smart devices because IoT could not be defined without smart sensors. We can consider our solution as the first authentication solution that can deal with the IIoT and, hence, with the industry 4.0. We can talk about Industrial Linux, which enables distributed IIoT applications. It is possible, in the future, that we work on converting our implementation to a firmware so that it can be executed under ordinary sensor devices.

## 8. Security Considerations

Traditional security techniques are characterized by their important overhead in terms of computation and communication costs. Hence, these mechanisms are infeasible in the WSN based-IoT that contains sensor nodes with low processing and memory capabilities. Table 16 recapitulates the possible threats that can occur during the LC-DEX handshake and the security requirements that should be considered.

*Collision attack*: The channel frequency is not fixed arbitrarily by the nodes. We propose to implement a dynamic selection of the Frequency Hopping Techniques after defining the quality metrics [41]. Besides, the interference probability of the exchanged packets between the communicating HIP peers decreases considerably. The combination of this technique in addition to the minimization of the communication overhead, improved by our proposed solution LC-DEX, may decrease the collision attack.

*DoS attack*: The countermeasures against DoS attacks must be defined regarding the different networking layers. Since our work focuses on the link layer (exactly on the MAC layer), we discuss only the DoS attacks on the link layer. HIP DEX is designed to protect communications against DoS attacks that are due to the puzzle mechanism. More the puzzle is difficult, more the communications are protected against DoS attacks. Besides, the minimization of the processing overhead in LC-DEX decreases the probability of such attacks.

*Flooding attack*: The compression and decompression are performed on the 6BR the most near of the HIP peers except the edge router. An adversary tries to flood the 6BR on the edge of the IoT domain. In our implementation, we propose that compressed HIP packets are only forwarded by the edge 6BR if the target is in the external network with the use of the firewalls and Intrusion Detection System (IDS). For this reason, we firstly propose that all the sensor nodes in the internal WSN are running a robust IDS like SVELTE [42] and especially the IDS proposed in [43] designed for securing WSN 6LoWPAN networks based on RPL as it is the adopted routing-protocol on our work. The internal 6BR is charged with the compression/decompression of HIP packets. Hence, with this strategy with the performed computation overheads of LC-DEX, flooding attacks will be minimized in advantage.

*Data freshness*: We study this attack on two sides: (1) Null old message is being replied by an adversary using an old shared key; (2) The attacker stored old HIP packets and then replay them with the communicating peers as it is a legitimate node.

In (1), an attacker used an old key to initiate the connection with the HIP peers at the same time as the distribution of a new key for all nodes in WSN. In our solution, HIP DEX shared key is not distributed to the nodes in the IoT domain. The shared secret key is shared only between the nodes that initiate communication using HIP DEX in the handshake.

In (2), the HIP DEX used a two-factor authentication mechanism [5]. The Initiator stores a shared group key that will be used to encrypt credentials in the I2 packet. After reception of the I2 packet, the responder verifies that its puzzle challenge was encrypted with the shared and correct key. If it is the case, the handshake is continuing the process, else the connection is discarded. The attacker does not know the group key shared between the HIP peers and especially the private key. We could also propose to add a time counter, as a sequence number of packets, to ensure data freshness by implementing an algorithm for that in the correspondent HIP peers. In this case, we propose that the 6BR, which takes the rôle of the collaborator on the proposed distribution model, check for every incoming packet from the HIP peers that the sequence number is strictly higher from the last received sequence number. Elsewhere, the 6BR (collaborator) drops any received packets with a previously used sequence number.

*Man in the middle attack (MITM)*: The leakage of the digital signatures in HIP DEX can cause an MITM attack for the R1 packet. This attack is treated on the R2 packet with the state machine. We reinforce our solution by the configuration of the HIT Access Control List (ACL) in the initiator, the responder, the CTTP, and all network appliances (router, firewalls). ACLs define the legitimate list of HITs and HIs. In addition, we propose to add the password used to authenticate the responder and the CTTP (see Section 4.1.2) in the ACL rather than storing it on a database on the base station. Thus, HIP peers discard any packet if the sender’s HIT (or HI) is not assigned in the ACL. Lunching a link-layer attack can be lessened by the minimization of the frame length and of the communication overheads we worked on in this paper. This optimization on the communication between the initiator and the responder lets the attacker not having enough time to capture exchanged packets on the communication channel. In addition, the adversary can not hold the HIT’s responder, since we proposed to revoke it. Hence, the initiator, when receiving the R1 packet from the adversary, identifies that it is not from the legitimate responder and discards it. For more precision, an indicator must be communicated between the HIP peers to indicate the removal of the HIT’s responder so that a malicious packet containing a HIT value will automatically be discarded by the initiator.

*Node capture*: The collaborator 6BR, on the LC-DEX solution, has a crucial role and if an attacker succeeds to attack it by a node capture attack, the hole network is damaged. Hence, we propose to not fix a collaborator for eternity. The collaborator on an IoT HIP domain changes dynamically in a short certain period. So, if an attacker has access to the collaborator, he loses authority on it after a very short period.

## 9. Results and Discussion

HIP DEX is the most encouraging candidate of the authentication or bootstrapping processes in IoT. Since it is more legitimate to optimize an IoT-designed protocol than working on existing Internet standards, our effort focuses on the quick authentication process to access a WSN integrated into IoT based on HIP DEX protocol. Authentication in IoT, which is composed of hundreds of devices, must have a lower cost overhead in terms of the computation and communication of exchanged packets. Indeed, we worked on minimizing the energetic costs of the authentication based on the HIP DEX protocol as a keying material. Our contribution is around three axes: the first one is the compression of the HIP DEX packets’ header with adaptation to the 6LowPAN packet header. The second one is about the optimization of the number of exchanged messages during the handshake. The last one consists of a novel solution that implements HIP DEX’s handshake in opportunistic mode. The proposed compression model, presented in Section 3.4, contributes with an efficient minimization of the energetic communication and computational costs comparing with existing works. The optimization of the handshake process concerns the avoidance of two signaling packets (NOTIFY and CLOSE_ACK). We are not the first to propose the compression technique of the HIP protocol, but we are the first to propose such a minimal compression header. In this section, we discuss and compare previous HIP solutions with LC-DEX. Slimfit [17] cannot be suitable for lossy IoT networks because it does not deal with the minimization of the communication overhead that we consider an obstacle on the real-time application on the IoT. In contrast, LC-DEX is considering this issue and shows better results in communication energy cost compared with previous works. D-HIP [19] and HIP-TEX [44] are not optimal solutions that can be implemented on IoT solutions because they increase the communication overhead due to the increase of the exchanged messages caused by the integration of the proxies selection on the distribution model during the authentication process. Security features in LHIP [16] are maintained and communicated over the exchanged messages but ignored by the pairs. LHIP is weak because it omits primordial security mechanisms as mutual authentication and key exchange. The authors of P-HIP propose the computation of unique pairwise keys when the device moves from session to session or changes from the network. P-HIP [36] provides the minimization of the communication cost by the use of minimal certificate size by the implementation of the ECQV. However, we think that optimization in minimizing the computational overhead on a constrained designed protocol such as HIP DEX is suitable than working on HIP BEX (the same case for CD-HIP [18]). P-HIP is suitable only for applications that are implemented over User Datagram Protocol (UDP) on the transport layer. UDP indeed is less consuming of energy in communication because it is quicker than Transport layer protocol (TCP) but TCP is more secure and guarantees the integrity of exchanged data that constitutes a critical requirement for sensitive data as those sent by a patient’s medical sensor connected to a dedicated health monitoring system. In addition, P-HIP is suitable only for real-time applications in IoT with fix and regular data traffic. P-HIP is very weak against Distributed Denial-of-service (DDoS) attack and exactly the DrDOS (Distributed Reflection Denial of Service) that is based on the spoofing UDP parameter when the application uses UDP. To be used, E-HIP [22] must be added to all devices in the target network and is not compatible with other versions of the HIP protocol. E-HIP is limited for IoT solutions that define the responder as a powerful server and can not be defined for a T2T architecture where both the initiator and the responder are constrained nodes. Both compression and optimization deal with the improvement in minimizing the overall energetic costs of sensor nodes. Another benefit of adopting the compression of packets is that the number of fragments, to be sent, decreases comparing with uncompressed handshake packets and as consequence the probability of packets’ lost decreases. We believe, it is natural that the cryptographic-key management must be lightweight and do not have a significant overhead since the number of communicating nodes in IoT is very high. We also demonstrated by real experimentation the efficiency of LC-DEX over multi-hops architectures, and we consider LC-DEX efficient WSN’s authentication solution for IoT. It is worth mentioning that our solution software was programmed to support both IPv4, IPv6, and 6LowPAN architecture. LC-DEX presents a novel security association establishment with a HIP DEX opportunistic mode. Opportunistic mode combined to the alleviation of the scalar multiplication of the key generation deal with an excellent energy consumption minimization that could be ameliorated in advantage.

## 10. Conclusions and Future Works

In this paper, we presented an efficient authentication solution based on the HIP DEX protocol as a keying material, CoAP for the application layer, and RPL in the routing process. It is an End-to-End establishment security solution between HIP-IoT devices feasible for T2T and M2M architecture. The proposed solution is based on an optimal compression HIP header with the support of an extension of 6LoWPAN compression. HIP DEX uses ECDH to minimize the cryptographic computation overhead during the authentication mechanism. Hence, we contribute to minimize the overall handshake transmission time with HIP DEX protocol, and we demonstrate that we improved the detailed delays of the total transmission delay comparing with previous and recent works based on HIP solutions and other existing solutions. The obtained results prove an excellent minimization regarding the energy communication and computational costs. Our novel distribution model based on the HIP DEX opportunistic mode and the delegation of the heavy scalar multiplication to compute the session keys are the strength of LC-DEX that contribute to secure the communication over the IoT based-WSN architectures with a minimal energy consumption. For future work, we assume that the integration of virtual abstraction of the Software Defined Networking in the definition of the WSN connected to IoT is an excellent future research field that deal with the improvement of the energy consumption in the constrained environments. Our solution can be expanded in the Industrial IoT solution like Deep-Green [28] to deal with the security requirements of the networking access of IoT devices for architectures based on dispersed computing that extends the existing computing model joint the optimization of computing resources in correlation with network resources.

## Figures and Tables

**Table 1:** Comparison between HIP based authentication solutions. (+) indicates high and (-) indicates low. Com.: Communication, Cpt.: Computational, F.T.: Fault Tolerance.

HIP Solution	Layer	Technique	Com. Cost	Cpt. Cost	Translation in the 6BR	DoS Attack Resistance	F.T.
LHIP [16]	HIP Layer	Omitting public key cryptography	+	-	May be required	Very weak	- -
E-HIP [22]	HIP layer	Removing messages and parameters	+	++	May be required	Supported	- -
HIP DEX [5]	HIP layer/Data link	Assumption of ECC Diffie-Hellman method	-	++	May be required	Supported	-
D-HIP [19]	HIP layer	Distribution messages	++	- -	Required	Server only	- -
Slimfit [17]	HIP layer	Messages compression	-	+	May be required	Server only	-
HIP DEX + AMIKEY [23]	HIP layer	Lightweight key management	++	+	May be required	Server only	+
HIP DEX [8]	HIP layer/ Data link	HIP DEX implementation in WSN	-	- -	Required	Supported	+
CHIP [21]	HIP layer	Compression	-	- -	Required	Supported	+
HIP DEX [8]	HIP layer/Data link	HIP DEX implementation in IoT	-	- -	May be required	Supported	+
CD-HIP [18]	HIP layer	Compressed and Distributed HIP BEX	++	++	Required	Supported	+
Bettoumi et al. [9]	Data Link	Compressed HIP DEX/6LowPAN header	-	-	Required	Supported	+

**Table 2:** HIP DEX header fields before and after proposed compression with LC-DEX.

HIP DEX Header Field	Length Before Compression (bits)	Length After Compression (bits)
Next header	8	0 or 8
Header length	8	0
Packet type	7	3
VER.	4	0
RES.	3	0
Fixed bits 0 and 1	2	0
Checksum	16	0
Controls	16	0
HIT_I	128	96
HIT_R	128	0
**Header length**	**40 bytes**	**Min header = 12 bytes, Max header = 13 bytes**

**Table 3:** A8 device specification.

Component	Description
CPU	ARM Cortex-A8 with M3 co-microcontroller
Speed	72 Mhz
RAM	256 MB
Transceiver	IEEE 802.15.4
Radio Bandwidth	250 kbps

**Table 4:** Used protocols on the network layers.

Layer	Protocol
Application	CoAP
Transport	TCP
Network	6LowPAN-RPL
Link	IEEE 802.15.4

**Table 5:** HIP DEX handshake packets’ sizes in bytes before and after proposed compression.

Packet	Packet’s Size before Compression	Packet’s Size after Compression [9]	Packet’s Size after Compression LC-DEX	Percentage Gain [9]	Percentage Gain with LC-DEX
I1	137	122	110	11%	20%
R1	228	213	201	7%	12%
I2	284	269	257	5%	9.5%
R2	193	178	166	8%	14%
**Overall packets sizes**	**842**	**782**	**734**	**7**%	**13**%

**Table 6:** Comparison of handshake packets’ sizes with other solutions.

Handshake	Messages	Exchanged Bytes	Percentage Gain of LC-DEX Comparing with Previous Works
E-HIP [22]	4	1536	52%
HIP BEX [34]	4	968	24%
PANA/EAP-TLS [35]	13	7061	90%
HIP-AKA [34]	8	936	22%
D-HIP [19]	4	3580	79%
HIP DEX + AMIKEY [23]	6	744	1.3%
Bettoumi et al. [9]	4	782	6%
LC-DEX	4	734	-

**Table 7:** Comparison of handshake’s transmission delays with [9] after the proposed compression.

Contribution	Transmission Time (s)
Bettoumi et al. [9]	0.025
LC-DEX	0.023
Percentage gain	8%

**Table 8:** Comparison of handshake’s transmission delay (ms) with P-HIP and Bettoumi et al. [9].

Packet	P-HIP	Bettoumi et al. [9]	LC-DEX
I1	133	3.9	3.5
R1	340	6.8	6.4
I2	418	8.6	8.2
R2	185	5.6	5.3
Overall transmission delay	1076	24.9	23.4

**Table 9:** Processing delays of handshake’s packets before and after proposed compression.

Architecture	I1 + I2 Processing Delays (s)	R1 + R2 Processing Delays (s)
WSN based-IPv4-IoT [9]	0.166	0.156
WSN based-6LoWPAN-IoT (Standard HIP DEX) [9]	0.188	0.168
WSN based-6LoWPAN-IoT (Compressed-HIP DEX) [9]	0.156	0.156
LC-DEX	0.146	0.146

**Table 10:** Processing delays’ (s) comparison with previous works.

	Results in [34]	Results in [35]	P-HIP [36]	Slimfit [17]	Bettoumi et al. [9]	LC-DEX
Initiator	0.95	1.799	0.3	1.88	0.156	0.146
Responder	0.79	0.729	-	1.895	0.156	0.146

**Table 11:** Overall E2E delays of handshake packets.

Architecture	Overall E2E HIP DEX Handshake’s Delay (s)
WSN based-IPv4-IoT	0.57
WSN based-6LoWPAN-IoT **(before compression)**	0.39
WSN based-6LoWPAN-IoT **(after compression)**	0.34

**Table 12:** Overall E2E delays of handshake packets.

HIP Solution	Handshake Latency Time (s)	Percentage Gain
P-HIP	1.076	70%
Bettoumi et al. [9] **(after compression)**	0.34	6%
LC-DEX **(after compression)**	0.32	-

**Table 13:** Handshake Energy Communication Costs. Orange: results calculated from Bettoumi et al. [9] and green: LC-DEX’s solution.

Handshake Packets	1 Hop (mJ)		4 Hop (mJ)		8 Hop (mJ)	
4 packets	1.729	1.62	1.959	1.85	2.267	1.26
6 packets	2.11	1.95	2.456	2.3	2.916	2.76
8 packets	2.998	2.79	3.459	3.25	4.074	3.86

**Table 14:** Communication and computational energetic costs of HIP existing solutions.

HIP Solutions	Computational Cost (mJ)	Communication Cost (mJ)	Total Energetic Cost (mJ)
Standard HIP DEX	13.365	0.329	**13.694**
Ben-Saied et al. [20]	3.43	94.55	**97.98**
D-HIP [19]	15.17	86.59	**101.76**
C-HIP [18]	63.622	47.186	**110.808**
CD-HIP [18]	14.93	52.16	**67.09**
Slimfit [17]	1.286	not indicated	-
Bettoumi et al. [9]	4.182	1.729	**5.911**
LC-DEX	3.931	1.62	**5.551**

**Table 15:** Memory consumption of LC-DEX software.

Extension	RAM (KiB)	Memory Percentage Use
Linux OS	7864	-
Initiator (I1 + I2)	8140 (+276)	3%
Responder (R1 + R2)	8136 (+272)	3%

**Table 16:** Security requirements.

Attack	Layer	Scenario	Defense
Collision attack	Link	A collision is triggered by the usage of the same channel frequency by the HIP peers.	HIP communicating peers should implement a solution to avoid collision attacks.
Denial of Service (DoS) attack	Link	An attacker may produce a collision when transmitting the signaling CLOSE_ACK packet. A bombardment in transmitting repeatedly such resulting collision has a costly resource consuming on the victim node.	HIP peers must be protected against DoS attack.
Flooding attack	Transport	An adversary try to flood the edge 6 BR to interrupt the communication between the peers.	Compressed HIP packets should be protected against the flooding attack on the 6LoWPAN edge router.
Data freshness	Link	An adversary lunch a replay attack using an old shared key at the same time when a new key is being distributed to the nodes. The adversary stores exchanged HIP messages and then initiates the communication with the peers.	HIP peers do not receive new keys from an external appliance. HIP peers should be able to detect malicious packets during the handshake.
MITM	Link	An attacker can store the HIP packets (I1, R1, I2, R2) and then replay a packet with the HIP peer as it is the legitimate target.	The exchanged HIP packets must be protected against MITM attacks.
Node capture		An attacker may lunch node capture attacks on the gateways and get cryptographic keys and add other malicious nodes in the network as legitimate ones.	A defending strategy must take place.

## Data Availability

The data presented in this study are available on request from the corresponding author. The data are not publicly available because it is the subject of a work in progress.
